# A secure blockchain framework for healthcare records management systems

**DOI:** 10.1049/htl2.12092

**Published:** 2024-10-09

**Authors:** Mahmoud Ahmad Al‐Khasawneh, Muhammad Faheem, Ala Abdulsalam Alarood, Safa Habibullah, Abdulrahman Alzahrani

**Affiliations:** ^1^ School of Computing Skyline University College University City Sharjah Sharjah UAE; ^2^ Jadara University Research Center Jadara University Irbid Jordan; ^3^ Department of Computing (Innovations & Technology) University of Vaasa Vaasa Finland; ^4^ College of Computer Science and Engineering University of Jeddah Jeddah Saudi Arabia; ^5^ Department of information Systems and Technology College of Computer Science and Engineering University of Jeddah Jeddah Saudi Arabia

**Keywords:** Blockchain technology, design science, electronic healthcare records

## Abstract

Electronic health records are one of the essential components of health organizations. In recent years, there have been increased concerns about privacy and reputation regarding the storage and use of patient information. In this regard, the information provided as a part of medical and health insurance, for instance, can be viewed as proof of social insurance and governance. Several problems in the past few decades regarding medical information management have threatened patient information privacy. In intelligent healthcare applications, the privacy of patients' data is one of the main concerns. As a result, blockchain is a severe necessity as it can enhance transparency and security in medical applications. Accordingly, this paper uses the design science method to propose a secure blockchain framework for healthcare records management systems. The proposed framework comprises five components: a blockchain network, smart contracts, privacy key management, data encryption, and integration with healthcare information technology. In the proposed framework, healthcare organizations can manage healthcare information securely and privately. Additionally, a secure storage system for electronic records is proposed to meet these organizations' needs. It provides security and privacy for healthcare organizations, especially when managing healthcare information, and also proposes a secure storage system for electronic records to meet the needs of the organizations.

## Graphical abstract

The paper discusses the increasing privacy and reputation concerns related to electronic health records in recent years and proposes a secure blockchain framework for healthcare records management. The framework includes components such as a blockchain network, smart contracts, privacy key management, data encryption, and integration with healthcare information technology, aiming to enhance transparency and security in managing healthcare information.

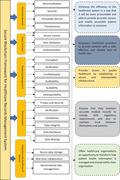

## 1 INTRODUCTION

Today, many healthcare, transportation, and financial services applications use blockchain technology. This technology has become increasingly urgent and necessary in recent years. The blockchain, or blockchain technology, is a distributed, public ledger used for recording transaction information, as well as tracking assets, which is secure, immutable, and distributed by way of peer‐to‐peer networks of computers rather than a centralized authority, now that the centralized authority has ceased to exist [1]. A company's assets can be tangible, such as a house or money, or intangible, such as patents or copyrights. Usually, a blockchain consists of a series of ordered records organized in a block structure. Hashes (digital fingerprints or unique identifiers) uniquely identify data blocks and time‐stamped batches of the latest transactions. Using this design, every block is linked chronologically and referred to as a blockchain. Since every block after the changed block must be changed at the same time, changing one of the blocks in the middle of the chain is almost impossible. Owing to this mechanism, the blockchain network data are immutable.

A healthy life is the foundation for a successful and happy life, and medical technology has played a significant role in making life more fulfilling and enjoyable for people today. Technological advancements make it easier to decipher the problems affecting our health. The healthcare industry manages many medical records about patients, doctors, and pharmaceuticals to offer better health care. Any healthcare organization faces the challenge of protecting these data from unauthorized users. Disclosing healthcare data may reveal a patient's identity and ailments, making them more sensitive. A healthcare organization that stores large amounts of medical data needs effective security mechanisms in this regard [2].

In many industries, blockchain technology has helped advance and improve efficiency [3]. This technology can record all events experienced by a product or subject from its conception until its present state. It has a variety of applications, such as checking the freshness of groceries, confirming the authenticity of art, and verifying land ownership. Blockchains also allow for smart contracts, which are pieces of code that are automatically executed based on predefined conditions.

**FIGURE 1:**
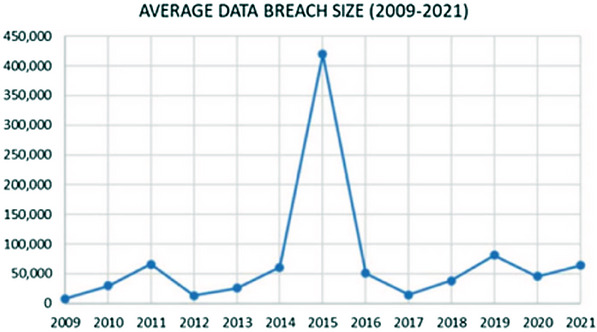
Data violations from 2009 to 2021 [6].

Healthcare is the industry that suffers from the most data breaches regarding the security of personal information [4]. Several healthcare practitioners have reported that between 2005 and 2019, there was a significant increase in the number of people impacted by healthcare data breaches. For instance, in 2018, there was at least one daily breach of health insurance records, as reported by the Health Insurance Portability and Accountability Act (HIPAA). The healthcare records of more than 59% of the US population have been compromised as a result of data breaches in the period between 2009 and 2018. There has been a significant increase in healthcare data breaches in 2015, with over 113.2 million records being exposed, stolen, or improperly shared with unauthorized individuals [5]. Figure 1 shows the data violations from 2009 to 2021 based on the number of data breaches.

A blockchain concept first appeared in the Bitcoin whitepaper to address the double‐spending problem without needing a trusted third party, such as a bank or a financial institution [7]. Decentralized currency and peer‐to‐peer electronic cash applications are among the specific functionalities of the first public blockchain behind Bitcoin. Therefore, Bitcoin's blockchain was difficult to customize with script or other scripting systems. The Ethereum blockchain platform, created by Vitalik Buterin [8], includes a Turing‐complete programming language built into it for writers of intelligent contracts. A few codes can be used to implement currencies, identities, and reputation systems on Ethereum [5]. After the Ethereum platform was introduced, people realized the value of blockchain applications and began researching alternative applications based on blockchain. The success of blockchain increased with the development of new blockchains and software stacks to build new blockchain technologies. In contrast to the internet era, where value was captured in the application layer, the technology has snowballed with wide adoption and investments. Consequently, there is a need to formalize the definition and categorization of blockchain technology for academics and industry. Scalability has become an urgent issue with blockchain‐based cryptocurrencies [9]. Having numerous transactions processed at a given time is one of the keys to scaling blockchain, and this low transaction throughput has been known for some time.

**FIGURE 2:**
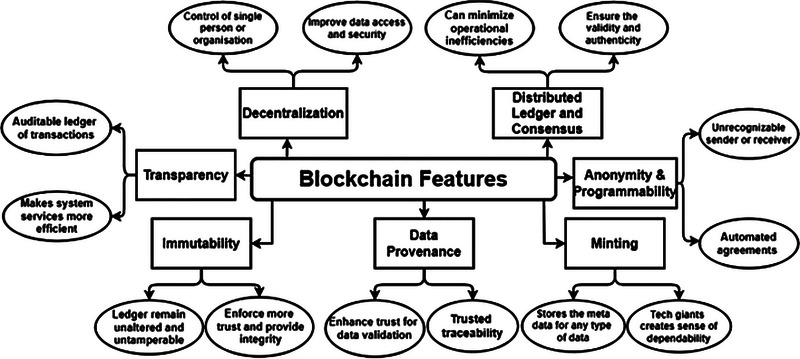
Features of blockchain technology [10, 11].

In recent years, according to [10, 11] blockchain technology has gradually become significant because of its various features, as illustrated below and in Figure 2.

1. **Decentralization**: Using these technologies, you can keep your assets in a network and not have them governed by the oversight or control of just one individual or an organization. Using algorithms to run the system, you have no chance of being scammed out of anything, as people cannot cheat you. No one can use blockchain technology for their own gain. However, in most contemporary organizations and facilities in the healthcare sector, this has been done mainly through centralized systems, i.e. a single body holds too much power over the rest.
2. **Transparency**: Data transparency can aid in creating an auditable, complete, and accurate ledger of transactions, which can be used in the healthcare industry. Currently, there is no solution to manage healthcare data in a way that simultaneously assures transparency, privacy, and security. Additionally, blockchain technology can also be used to facilitate greater transparency by ensuring the security of data and enabling authorized parties to have access to healthcare data, with mechanisms for encryption and control able to achieve that.
3. **Immutability**: A great advantage of blockchain is that such a ledger can remain unaltered over the long run, and that is its most remarkable feature. In terms of auditing, it can redesign and restructure the process to be more efficient, quicker, and cost‐effective in the long run. Because of this feature, a user can almost not modify or delete any information on the network. Cryptographic hashing is a method that can be used to achieve this feature.
4. **Data source for the healthcare industry**: To increase public trust in using health data, all relevant information regarding the creation, access, and transfer of health data must be provided to improve data provenance. The capability of tracking changes in data from its creation to its present state ensures this type of security. With blockchain technology, timestamping takes place through a procedure involving evaluating the hash values of the provenance record to confirm a reliable ledger of all legal transactions that have been maintained. Once a hash value has been determined, it is forwarded to consensus nodes for verification.
5. **Minting**: Blockchain can, in essence, solve various problems related to manipulation. As a result, people and corporations in the West feel that banks and global technology firms, such as Google and Meta, could be more dependable and accountable if they use blockchain [12]. Blockchain has a chance to be an up‐and‐coming technology not only in countries where mining is still prevalent but also in countries that have not yet reached a level of adoption.
6. **Secrecy and programmability**: Blockchains are distinguished by their anonymity and programmability. In contrast to anonymity, intelligent contracts with programmability can automate transactions by enabling new regulations to be enacted, while anonymity assures that the individuality of senders and beneficiaries remains anonymous during the transaction. As a result of agreements between buyers and traders, smart contracts are referred to as self‐executing programs.
7. **Scattered ledger and agreement**: It is more than likely that blockchain can present several advantages in the future as it combines fundamental technologies such as distributed ledgers and consensus techniques. Consensus is a decision‐making procedure for the network's active nodes when millions of nodes validate transactions simultaneously. This algorithm is crucial to the smooth functioning of the system. A distributed ledger system (DLT) makes the process transparent and dependable by ensuring anyone with the necessary access can monitor the ledgers in real‐time.

Therefore, there is no doubt that electronic health records are an essential component of any health organization. Several concerns have been raised regarding the storage and use of patient information over the past few years, mainly regarding privacy and reputation. For this reason, it is possible to see the information provided as part of a medical or health insurance policy, for instance, as a confirmation of social insurance and good governance. As a result of several problems regarding medical information management over the past few decades, the privacy of patient information has been at risk. One of the key concerns when it comes to intelligent healthcare applications is ensuring the confidentiality of patients' data.

Accordingly, this paper uses the design science method to propose a secure blockchain framework for the healthcare records management system. The proposed framework involves five components: a blockchain network, smart contracts, privacy key management, data encryption, and integration with healthcare information technology. Developing a secure blockchain framework for healthcare records management offers numerous contributions that can improve patient information security, traceability, privacy, interoperability, and data accuracy. By leveraging the features of blockchain technology, healthcare providers can overcome the limitations and challenges of traditional record management systems, paving the way for a more efficient, secure, and patient‐centred healthcare system.

A secure blockchain framework for healthcare records management systems offers numerous contributions that can significantly improve the efficiency and security of managing medical records. These contributions include enhanced data security and privacy protection, seamless interoperability and sharing, and data accuracy and integrity. By embracing blockchain technology, healthcare organizations can unlock the full potential of electronic health records and empower patients and other stakeholders with secure and easily accessible medical information.

The rest of the article is prepared as follows: Section 2 discusses the existing literature. Section 3 introduces the methodology and development processes. Sections 4 and 5 present the discussion and conclusion, respectively.

## 2 LITERATURE REVIEW

Several studies have been introduced in the literature about the healthcare records, blockchains, and the role of blockchains in these records. This section focuses on both sides of healthcare records and blockchain technology.

The authors in [13] suggested a framework based on blockchain to secure the control of personal health information being shared between healthcare providers. This is to offer better care for the patient. The suggested framework contains a mechanism for controlling the data to reduce risk.

Using Blockchain technology, the authors of [14] showed that it can effectively transform the Electronic health records (EHR) system and that there could be a variety of solutions to address these issues using blockchain technology. The authors then demonstrated a blockchain‐based framework for the healthcare sector. This framework provides granular access rules for users of the proposed framework to maintain secure and efficient storage of patient‐related health records. A scalability framework for using blockchain technology was also presented as part of their framework.

The authors proposed a new architecture [15] to enable safe sharing, which in turn allowed stakeholders to gain the maximum benefit from the unique properties of blockchains. It was found that by promoting data authenticity, the proposed design increased the security of devices. A consensus‐based transaction system was used to build an interoperable and efficient system for executing transactions using the consensus principle. To enhance cooperation among nodes across blockchain networks maintained on the third level of the system, the system was built on the top of a tripartite design on several levels, including a blockchain framework, application middleware, and smart contracts, all of which were part of the tripartite design.

According to [16] IoT healthcare networks should be shared for patient observation. Their proposed design effectively sends the data to microcontrollers and stores it in MySQL databases by separating it from intelligent energy‐efficient biosensors. For health experts and patients worldwide, primary data and investigative information must be checked, gathered, analysed, and recorded efficiently. However, the authors did not explore security features in the proposed solution.

In [17], an artificial system was proposed to improve diagnostic accuracy and quality using a blockchain paradigm. The emerging Artificial Health System will be able to administer treatments following the models provided to the patient by creating and understanding the complementary components between humans and simulated clinicians.

The authors in [18] proposed an economical, cost‐effective solution to the problem of health sensor networks. Several features in the proposed software include encrypted messages, medical devices, and remote centres. In addition, the proposed software was integrated with a new approach to applications, online video sharing, messaging, automatic prescriptions, and automatic messages for medical devices.

In [19], the authors investigated the benefits that modern blockchain‐based solutions provide for protecting medical data in cloud‐based and non‐cloud‐based scenarios. They also compared blockchain and conventional methods in this regard.

The researchers in [20] They proposed a permissioned blockchain solution based on the Hyperledger Fabric framework, which can store and share electronic healthcare records. The proposed system has several advantages, including enabling patients to have complete control over their electronic medical records and enabling doctors to review them and add new EHR data using methods such as grant and revoke access.

To address the challenges that concern the Internet of Medical Things in a secure and immutable manner, the authors in [21] proposed a blockchain‐based framework based on the principle of immutability, as part of the proposed architecture, hospitals and users can perform numerous healthcare‐related operations securely and confidently through a lightweight private blockchain network.

A novel patient‐centred blockchain‐based EHR management system was developed by [22], which allows health records management among multiple stakeholders without centralized infrastructure. This system provides patient privacy without a centralized infrastructure and allows or revokes access to or views patient records.

As a way to ensure that EHR sharing among different electronic healthcare systems is secure, the researchers in [23] proposed a blockchain architecture that relies on Proof of Stake (POS) cryptography consensus mechanisms and the Secure Hash Algorithm (SHA256), which authenticates the user identity using blockchain technology.

Using blockchain technology, the authors in [24] proposed a method for healthcare data sharing to be secured under the control of patients. Blockchain technology and the Interplanetary File System are incorporated as part of this scheme.

An inter‐healthcare EHR exchange based on blockchain technology was proposed in [25]. The proposed architecture identifies and blocks malicious actions from outsiders and insiders on stored and shared EHRs. In addition, it can generate standard formats that diverse healthcare systems can easily understand to confirm the integrity and reliability of EHR requests and replies.

Therefore, it can be said with complete certainty that electronic health records are integral to every healthcare organization today. Several concerns have been raised over the past few years about the storage and use of patient information. These concerns are mainly regarding the hospital's privacy and reputation. In this case, it is possible to see the information provided as part of a health insurance policy, for instance, as an indication of social insurance and responsible governance, in addition to the information provided as part of a medical insurance policy. The privacy of patient information has been at risk for over a decade, mainly due to several problems related to medical information management over the past few decades. One of the key concerns when it comes to intelligent healthcare applications is ensuring the confidentiality of patients' data.

## 3 METHODOLOGY AND DEVELOPMENT STEPS

**FIGURE 3:**
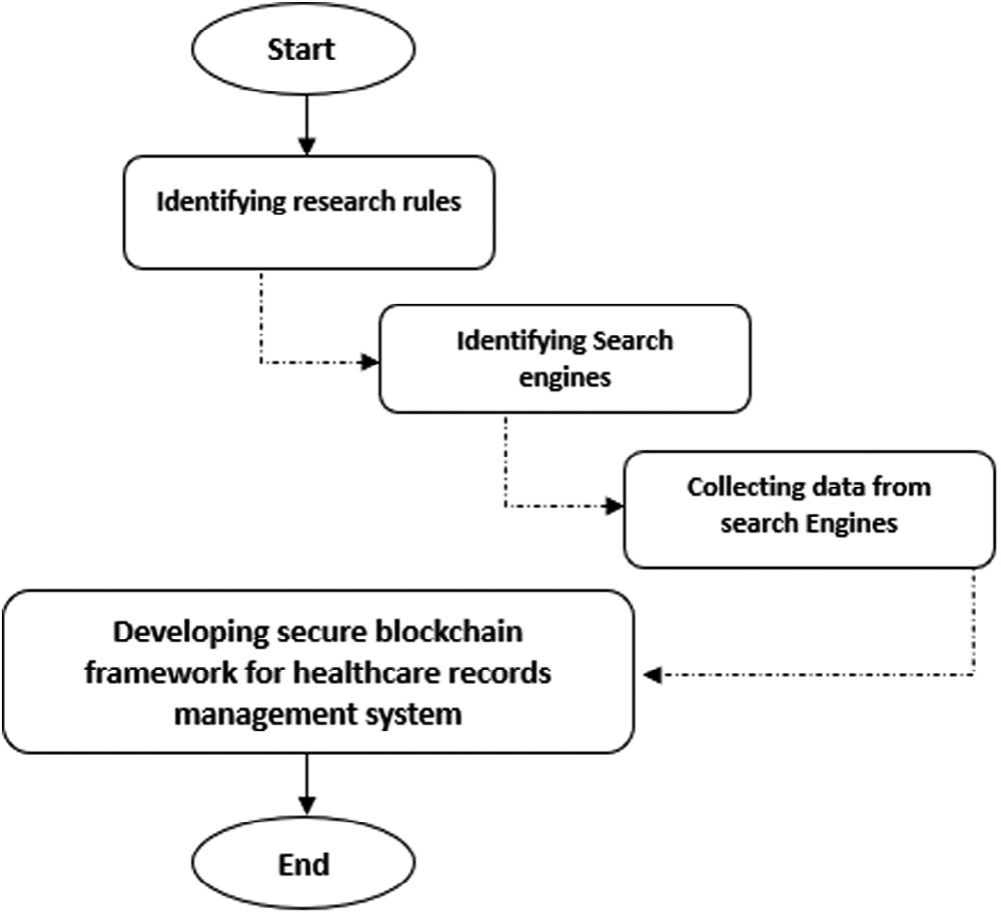
Research methodology and development processes.

This section explains the methodology and development steps used in this study. The design science methodology was used in the present paper to develop the secure blockchain framework for healthcare records management systems. This methodology was used to create the component's structure and the constituent elements of the component, which are used to determine the component's structure and the constituent elements of the component itself [26]. Figure 3 shows the methodology adopted in this research.

1. Identifying searching rules: To narrow the search for the article, the author used a set of keywords relevant to the topic, such as “Electronic healthcare records” and “Blockchain technology.” In addition, the search was limited to articles published in journals or conferences in English between 2015 and 2023.
2. Identifying search engines: This study determined that five search engines should be used to search for required data. The search engines were Scopus, IEEE Xplore Springer Link, Web of Science, and Google Scholar.
3. Collecting data from the search engines: In this process, relevant articles published about blockchain technology for EHRs were collected using the search rules we defined in Step 1. Initially, the titles and abstracts of the articles selected for review were screened. After that, the conclusions of the articles and the entire article's content were reviewed.
4. Developing secure blockchain framework for healthcare records management system: Various phases were involved in developing a secure blockchain framework for managing the healthcare records. This part of the paper will deliver a thorough outline of those phases. The following is a list of the phases provided by the developed framework:
  1. Reducing the danger: Blockchain technology safeguards healthcare records using cryptography. As a result, data breach and unauthorized access risks are reduced, resulting in fewer data breaches.
  2. Avoiding modifying healthcare records: A decentralized blockchain architecture confirms that no one can change healthcare records. A blockchain audit trail is provided to assist in verifying the accuracy of the data and to prevent allegations of deception by preventing deception from happening.
  3. Authorising Patients: Patients can own and control their healthcare records. They will be able to log on, access their records, and share them faultlessly with the healthcare providers of their choice at any time.

**FIGURE 4:**
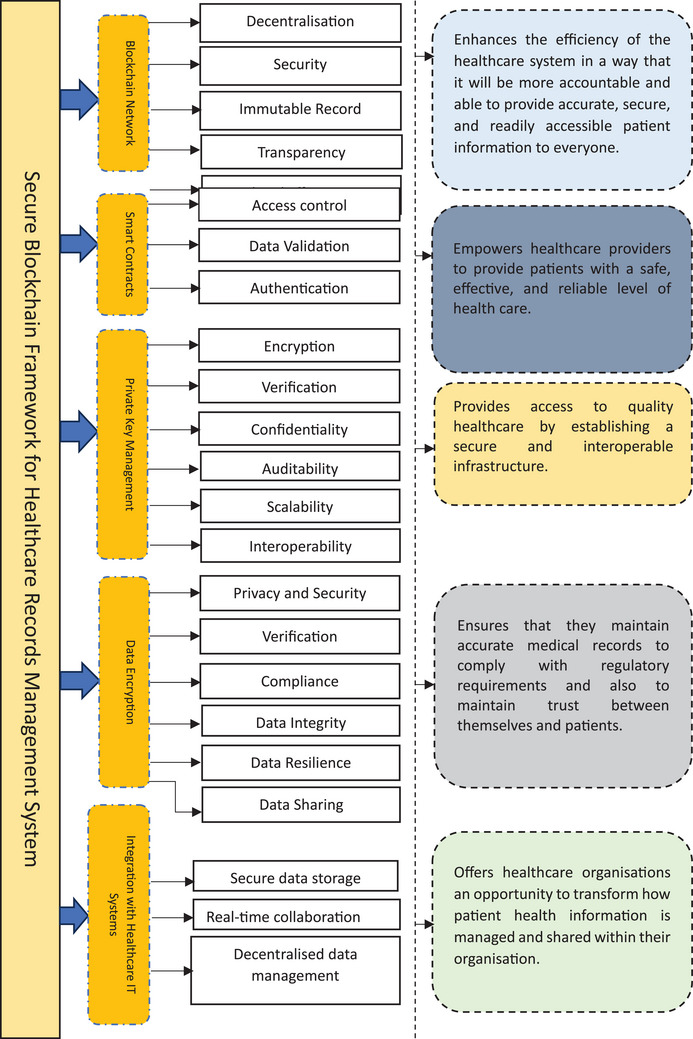
Secure blockchain framework for healthcare records management system.

Therefore, the developed secure blockchain framework for healthcare records management system comprises the subsequent vital elements (see also Figure 4):

1. **Blockchain network**: In the blockchain infrastructure, nodes form a decentralized network that makes up the chain. The nodes are responsible for ensuring the integrity of the network by verifying transactions and adding new blocks to the chain daily. A blockchain network offers several benefits, including:
  ✓ Decentralization: Since it is a decentralised network and not controllable by a single entity, it is impossible for any authority to control it or for a single point of failure to occur. This enhances both the security and reliability of the system.
  ✓ Security: As blockchain networks make all transactions encrypted and immutable, their security and reliability cannot be compromised. This means they are resistant to fraud and tampering.
  ✓ Immutable record: Once a transaction has been recorded on the blockchain, it cannot be erased or altered. Therefore, every transaction is recorded in the database in a way that ensures maximum transparency and security.
  ✓ Transparency: The network ensures that all transactions are transparent and accountable since every node has access to every transaction taking place, making it possible to track all transactions as they are being completed.
  ✓ Speed and efficiency: A blockchain network is a network of nodes managed by a consensus model that processes and confirms transactions in real‐time, resulting in a highly efficient and fast system.
2. **Smart contracts**: Smart contracts can automate a wide range of business processes, resulting in greater efficiency and productivity for businesses using blockchain technology. Executing contracts automatically on the blockchain is possible based on certain conditions or events. In addition to their main advantage, self‐executing contracts can simplify and automate complex processes. These processes typically require manual participation to be completed, and human involvement is likely to result in errors and delays. No manual intervention is necessary with smart contracts since their code can be incorporated directly into the software. Smart contracts offer several other advantages, including streamlining and automating administrative tasks. Using blockchain makes it possible to encode contract rules and conditions and automatically execute them as soon as they are met. Therefore, no human intermediaries are permitted, reducing the possibility of fraud and errors. Smart contracts comprise three applications:
  ✓ Access control: Smart contracts can be used in various applications for access control; this is one of the most used applications of these contracts. Organizations can grant specific groups of people access to different resources by putting in place self‐executing contracts that will enable them to specify in advance predefined rules and permissions for granting access to these resources to specific individuals based on specific criteria.
  ✓ Data validation: A smart contract can also verify the integrity and authenticity of data, eliminating the possibility of those data colliding. Blockchain technology could be implemented in any manner, thereby allowing the users of this type of contract to ensure that the data they are working with has not been altered, tampered with, or modified in any way by users.
  ✓ Authentication: Smart contracts offer a convenient and secure authentication solution. As a way of establishing the authenticity of transactions, smart contracts are highly efficient and safe methods. Blockchain technology is being used to create unique identification codes for users and devices by using these contracts, eliminating the need to use usernames and passwords for logins and other authentications that were formerly needed. In addition, this will increase the security of systems and ensure that different systems and programs can interact seamlessly with each other simultaneously, improving both their effectiveness and security.
3. **Private key management**: A private key management system must be used when managing healthcare records to ensure that every record is safe, secure, confidential, and authentic. This will enhance patient safety and compliance with government regulations. The private key management provides the following features for the healthcare records systems:
  ✓ **Encryption**: Private keys enable the encryption of healthcare records, ensuring that only authorized parties can access and decrypt the data.
  ✓ **Verification**: These keys help users verify healthcare records' authenticity and integrity.
  ✓ **Confidentiality**: The decentralized nature of blockchain technology ensures that healthcare records are stored securely across multiple nodes, enhancing confidentiality and reducing the risk of data breaches.
  ✓ **Auditability**: Private key management enables transparent auditing of healthcare records, providing accountability and traceability.
  ✓ **Scalability**: Private critical management systems can be easily scaled to accommodate the growth of an extensive healthcare record system.
  ✓ **Interoperability**: These systems can be easily integrated with other healthcare infrastructure, such as electronic health records systems, to ensure seamless and secure data transfer.
4. **Data encryption**: A data encryption process can be defined as a process through which a secret key or algorithm is used to scramble data so that they become unreadable and unintelligible for anyone who does not have a decryption key to decrypt and decode the data. The safety of patient data can be ensured by encrypting them to prevent unauthorized access, corruption, or modification of the data by healthcare organizations. A healthcare records management system that uses data encryption has the following benefits:
  ✓ **Privacy and security**: Data encryption guarantees patient data stays private, preventing illegal individuals from logging on or employing sensitive data.
  ✓ **Compliance**: Healthcare corporations must comply with actual data safety laws like HIPAA and GDPR. Data encryption helps organizations meet these governing constraints.
  ✓ **Data integrity**: No risk of data interfering or fraud will exist when data is encrypted during transmission or storage, as encryption ensures that the information stays unaffected.
  ✓ **Data resilience**: Blockchain frameworks often apply decentralized networks, making data encryption even more critical to safeguard patient data.
  ✓ **Data sharing**: Data encryption allows the secure revealing of persistent data among healthcare contributors and investigators without compromising isolation.
5. **Integration with healthcare IT systems**: A secure blockchain framework for EHR components requires integrating healthcare IT systems to ensure patient data's smooth functioning and integrity. Integration of blockchain technology into the healthcare industry is crucial for maximizing its potential. Healthcare professionals can seamlessly integrate blockchain technology into health IT systems. As a result of this integration, patient data can be seamlessly shared between hospitals, clinics, and laboratories. Using blockchain technology to ensure the security and confidentiality of patient health information, healthcare IT systems can leverage blockchain's decentralized and immutable nature. Encrypting and authenticating patient data with blockchain's cryptographic algorithms provide secure access to sensitive data for only authorized parties. In addition, blockchain integration with healthcare IT systems makes real‐time data sharing and collaboration possible. Healthcare professionals from various institutions can view and update patient records in real‐time, improving the coordination and continuity of the care given.

## 4 RESULTS AND DISCUSSION

This section aims to discuss the research results and the effects observed. Using blockchain technology, this study developed an EHR framework for healthcare records. This framework ensures the confidentiality, accuracy, and accessibility of EHRs. Using the framework, a healthcare records management system was developed to meet the needs of healthcare organizations. It provides encryption, controls access to patient data, and offers consensus algorithms for data security.

One of the main conclusions of this study is that blockchain technology has several advantages over traditional EHR systems. A decentralized infrastructure and cryptographic technique enhanced the security, immutability, and traceability of EHRs.

Blockchain technology has been shown to offer several advantages over traditional EHR systems, particularly in terms of security, immutability, and traceability.

✓ **Security**: Blockchain uses cryptographic techniques, such as hashing and public‐private key encryption, to ensure that data is secure and can only be accessed by authorized parties. Each transaction or data entry on a blockchain is encrypted and linked to the previous transaction, creating a secure chain that is resistant to tampering. This method of securing data has been extensively studied and demonstrated in various fields, including healthcare. For instance, Kumar and Tripathi [27] show that blockchain's encryption mechanisms significantly reduce risks associated with unauthorized data access and breaches compared to traditional systems.
✓ **Immutability**: One of the core features of blockchain technology is its immutability. Once data is recorded in a blockchain, it is nearly impossible to alter or delete it without altering every subsequent block, which would require consensus from the majority of nodes in the network. This immutability is particularly beneficial for EHR systems as it ensures that patient records remain unaltered over time, providing an auditable trail that enhances trust in the accuracy of medical data. Studies like those by Khan et al. [28] emphasize the effectiveness of immutability in preventing tampering of medical records.
✓ **Traceability**: Blockchain's decentralized nature enables real‐time tracking of data access and modifications. Each time a healthcare provider, insurance company, or patient accesses or modifies a record, the transaction is logged on the blockchain. This ensures that there is a clear and transparent history of all interactions with a patient's data, improving accountability and data provenance. Several studies, including those by Shahnaz et al. [29], demonstrate that blockchain's decentralized infrastructure enhances the traceability of healthcare data, allowing for better auditability and reducing fraud.

Security, immutability, and traceability are some challenges facing a traditional EHR management system. This could result in patient and healthcare staff safety risks related to the use of EHRs. However, it should also be noted that blockchain technology has several unique properties that can be used as a platform for managing electronic health records. This solution addresses all these concerns. Cryptography holds several advantages, one of which is its decentralized nature, which, in other words, does not allow data to be controlled by one individual or entity. This is one of the significant advantages of cryptography. Hackers and other cybercriminals will find it challenging to gain access to information if organizations use this distributed architecture. This way, data breaches will be less likely to occur because of this measure. Additionally, since blockchain is immutable, once an EHR record has been created or modified, it will never be able to be altered or deleted in the future. Consequently, the data should remain reliable and verifiable based on this characteristic and provide a comprehensive and transparent audit trail in the future. In addition to its traceability feature, blockchain technology offers several additional benefits for healthcare providers, such as tracking health records across multiple stakeholders, e.g. hospitals, clinics, and insurers. Moreover, this feature facilitates seamless collaboration between healthcare providers, ensures precision data, and reduces errors. Blockchain's most substantial advantages over traditional EHR management systems are security, immutability, and traceability. The integrity, accuracy, and confidentiality of EHRs can be assured by leveraging these properties, resulting in better patient outcomes.

Blockchain technology also employs advanced encryption techniques, such as asymmetric (public‐private key) encryption and hashing, which ensures that only authorized parties can access or modify healthcare records. These encryption techniques offer superior protection compared to traditional centralized systems, where a breach in the central database could expose a vast amount of sensitive data. In contrast, the decentralized nature of blockchain minimizes this risk by ensuring that even if a single node is compromised, the entire system remains secure. Research by Kumar and Tripathi [26] confirms that blockchain's encryption techniques provide enhanced security and privacy for healthcare data, greatly reducing the risk of data breaches.

Additionally, blockchain provides cost‐effective solutions by reducing the overhead associated with maintaining and securing large centralized databases. Traditional EHR systems often require high operational costs for data storage, system maintenance, and security protocols to prevent breaches. Blockchain, however, distributes data storage across a network of nodes, eliminating the need for a central authority to manage the system, thereby reducing the costs associated with centralized infrastructure. According to a study by Vidap et al. [30], the decentralized nature of blockchain leads to reduced operational and maintenance costs, as it requires fewer resources to secure and manage the data.

The security of blockchain systems is further enhanced by the immutability of records, where every transaction is recorded in a transparent and tamper‐proof manner. Unlike traditional systems where records could potentially be modified or deleted, blockchain ensures that once data is entered, it cannot be altered without network consensus. This immutable feature is a key factor in preventing fraud and unauthorized access, as demonstrated in a study by Mahajan et al. [31], which highlighted blockchain's role in protecting healthcare data from tampering.

Thus, by using encryption, decentralization, and immutability, blockchain technology is not only more secure than traditional methods but also provides a cost‐effective solution for managing healthcare records.

This study found several important implications for the healthcare industry. EHRs can significantly benefit from a blockchain framework to enhance their security, integrity, and accessibility. By improving quality and streamlining healthcare processes, patient safety can be enhanced. Furthermore, blockchain technology can help mitigate data breaches in healthcare records management, which are becoming increasingly prevalent. By decentralizing data distribution, blockchain ensures that unauthorized access or manipulation is less likely.

Moreover, blockchain frameworks for EHRs may reduce costs. Health organizations can also eliminate paper‐based records, reduce storage costs, and reduce errors associated with manual entry. Integrating blockchain in healthcare records management can improve interoperability between healthcare organizations. Blockchains allow for seamless data transfers between facilities, systems, and patients, reducing the inefficiencies and complexity of current data exchange practices.

## 5 CONCLUSION

Among the most critical components of any healthcare organization are electronic EHRs. Several privacy and reputation concerns have arisen due to concerns about the storage and use of patient information in recent years. Therefore, the information provided when dealing with medical insurance and medical health insurance, for instance, can be viewed as evidence of social insurance and governance that exist in society. During the past few decades, many problems have arisen associated with managing medical data, which has created some threats to the privacy of patient data. One of the main concerns regarding the security of patient information in innovative healthcare applications is ensuring patient data confidentiality is protected. As a result, using blockchain as a solution to this problem is an absolute necessity. Using blockchain technology, it is possible to achieve transparency and security in medical applications. Using the design science method as a basis for development, this paper presented a design science‐based framework for a secure blockchain system to manage healthcare records. The proposed framework comprises five components: the blockchain network, smart contracts, privacy key management, data encryption, and integration with healthcare information technology systems. The proposed framework considers the possibility of providing security and privacy for healthcare organizations when managing healthcare information. Also, a secure storage system for electronic records is proposed to meet the needs of these organizations. Future studies need to complete several areas of work, including implementing the developed framework in real‐world organizations so that its effectiveness and capabilities can be demonstrated.

## AUTHOR CONTRIBUTIONS

**Mahmoud Ahmad Al‐Khasawneh**: Conceptualization; methodology; writing—original draft. **Muhammad Faheem**: Conceptualization; methodology; writing—original draft. **Ala Abdulsalam Alarood**: Conceptualization; resources; validation; writing—original draft. **Safa Habibullah**: Methodology; validation; writing—review & editing. **Abdulrahman Alzahrani**: Conceptualization; methodology; validation.

## CONFLICT OF INTEREST STATEMENT

The authors declare no conflicts of interest.

## Data Availability

Data available on request from the authors.
